# Autonomic nervous system modulation in arrhythmias: mechanisms and therapeutic advances

**DOI:** 10.3389/fphys.2026.1905077

**Published:** 2026-09-01

**Authors:** Yilan Zhou, Yingxin Li, Jiani Qiu, Ying Bai, Shukun He, Jiawei Shi, Jingrong Jiang, Yun Yang, Jingyi Zhang, Haotian Gu, Jing Wang, Qiaofeng Jin, Tianshu Liu

**Affiliations:** 1 Department of Ultrasound Medicine, Union Hospital, Tongji Medical College, Huazhong University of Science and Technology, Wuhan, Hubei, China; 2 Clinical Research Center for Medical Imaging in Hubei Province, Wuhan, Hubei, China; 3 Hubei Province Key Laboratory of Molecular Imaging, Wuhan, Hubei, China; 4 Key Laboratory of Biological Targeted Therapy(Huazhong University of Science and Technology, Ministry of Education, Wuhan, Hubei, China; 5 School of Cardiovascular and Metabolic Medicine & Sciences, King’s College London BHF Centre of Research Excellence, London, United Kingdom

**Keywords:** arrhythmia, autonomic nervous system modulation, neuroregulation, sympathetic nervous system, vagal modulation

## Abstract

Arrhythmia is a clinically common cardiovascular disorder characterized by high incidence and a strong association with sudden cardiac death, posing a significant threat to patient survival and quality of life. The pathogenesis of arrhythmia is complex, and abnormal autonomic nervous system (ANS) modulation, as one of the causes, offers new perspectives and therapeutic targets for arrhythmia treatment. This review discusses the mechanisms by which abnormal ANS modulation leads to arrhythmias and strategies for treating arrhythmias through ANS modulation.

Arrhythmia is a prevalent cardiovascular disorder marked by a high incidence and a strong correlation with sudden cardiac death, thereby posing a significant threat to patient survival and quality of life. The pathogenesis of arrhythmia is intricate, and abnormal autonomic nervous system (ANS) modulation represents one of the contributing factors, offering novel perspectives and therapeutic targets for treatment. This review examines the mechanisms through which abnormal ANS modulation induces arrhythmias and explores strategies for managing arrhythmias via ANS modulation. Arrhythmia is defined as an abnormality in the frequency, rhythm, origin, conduction velocity, or sequence of cardiac impulses, which can result in severe conditions such as heart failure (HF), stroke, and even sudden death (Ko et al., 2025). In 2021, the global prevalence of atrial fibrillation (AF) and atrial flutter reached 52.6 million (Tan et al., 2025). Various factors can contribute to arrhythmias, including abnormal ANS modulation, structural heart abnormalities, and ion channel dysfunction (Herring et al., 2019). Among these, abnormal ANS modulation is a significant pathogenic mechanism of arrhythmia, providing new insights and therapeutic targets for its management. This article reviews the mechanisms of ANS modulation in the treatment of arrhythmias and discusses its potential clinical applications for ventricular arrhythmia (VA) and AF.

Building on previous reviews of device-based and surgical neuromodulation, this review additionally integrates the hierarchical neurocardiac axis with neuroimmune remodeling, emerging molecular and bioengineering interventions, and a structured appraisal of translational maturity. It distinguishes guideline-supported or clinically used strategies from observational rescue therapies and preclinical proof-of-concept approaches, while highlighting conflicting clinical findings and unresolved questions regarding safety, durability, and patient selection.

## 1 ANS and the pathogenesis of arrhythmias

The mechanisms through which abnormal autonomic nervous system (ANS) modulation contributes to arrhythmias are primarily linked to ANS abnormalities, immune cell infiltration, and inflammatory cytokines. These findings establish a theoretical basis and suggest potential therapeutic strategies for the prevention and treatment of arrhythmias via ANS modulation.

### 1.1 Physiological organization and electrophysiological regulation of the cardiac autonomic nervous system

The cardiac autonomic nervous system comprises central autonomic networks, intrathoracic sympathetic and vagal pathways, cardiac plexuses, and the intrinsic cardiac nervous system. These components regulate cardiac automaticity, conduction, contractility, and electrical stability through afferent, efferent, and local neural circuits (Ardell and Armour, 2016; Fukuda et al., 2015; Ajijola et al., 2025b).

Sympathetic activation acts mainly through norepinephrine and β-adrenergic receptors. It increases sinoatrial node automaticity, atrioventricular conduction, myocardial contractility, and intracellular calcium cycling. Parasympathetic activation acts mainly through acetylcholine and M2 receptors. It reduces cAMP signaling and modulates If, ICa,L, and IK,ACh. These effects slow nodal conduction and shorten atrial action potential duration and effective refractory period.

Autonomic innervation varies across cardiac regions. The sinoatrial and atrioventricular nodes are densely innervated. The posterior atrium, pulmonary veins, and ligament of Marshall also receive abundant sympathetic and parasympathetic inputs. These structures are closely associated with atrial fibrillation initiation and maintenance (Ajijola et al., 2025b; Zwanenburg et al., 2025).

Ventricular innervation shows regional and transmural heterogeneity. Sympathetic fibers are generally denser near the epicardium and decrease toward the endocardium. However, this pattern varies among species, developmental stages, cardiac regions, and disease states. SEMA3A and other axonal guidance molecules contribute to this distribution (Fukuda et al., 2015; Koppel et al., 2024).

From an electrophysiological perspective, sympathetic and parasympathetic modulations alter arrhythmia susceptibility. They do so by influencing automaticity, calcium handling, conduction velocity, action potential duration, and effective refractory period (Fukuda et al., 2015; Ardell and Armour, 2016; Ajijola et al., 2025b). Under pathological conditions, excessive sympathetic activation promotes calcium overload. It also induces RyR2-related diastolic calcium leak, delayed afterdepolarizations, and repolarization dispersion. Parasympathetic activation promotes atrial reentry. It does so by shortening the atrial effective refractory period and increasing spatial heterogeneity. When both branches are co-activated, they synergistically enhance triggered activity and reentrant substrates in the atria. This synergy facilitates the occurrence of atrial fibrillation (Ajijola et al., 2025b; Ardell and Armour, 2016).

### 1.2 Structural and functional abnormalities of the ANS

#### 1.2.1 Cardiac neural remodeling: a consequence of myocardial ischemia

**Figure 1:**
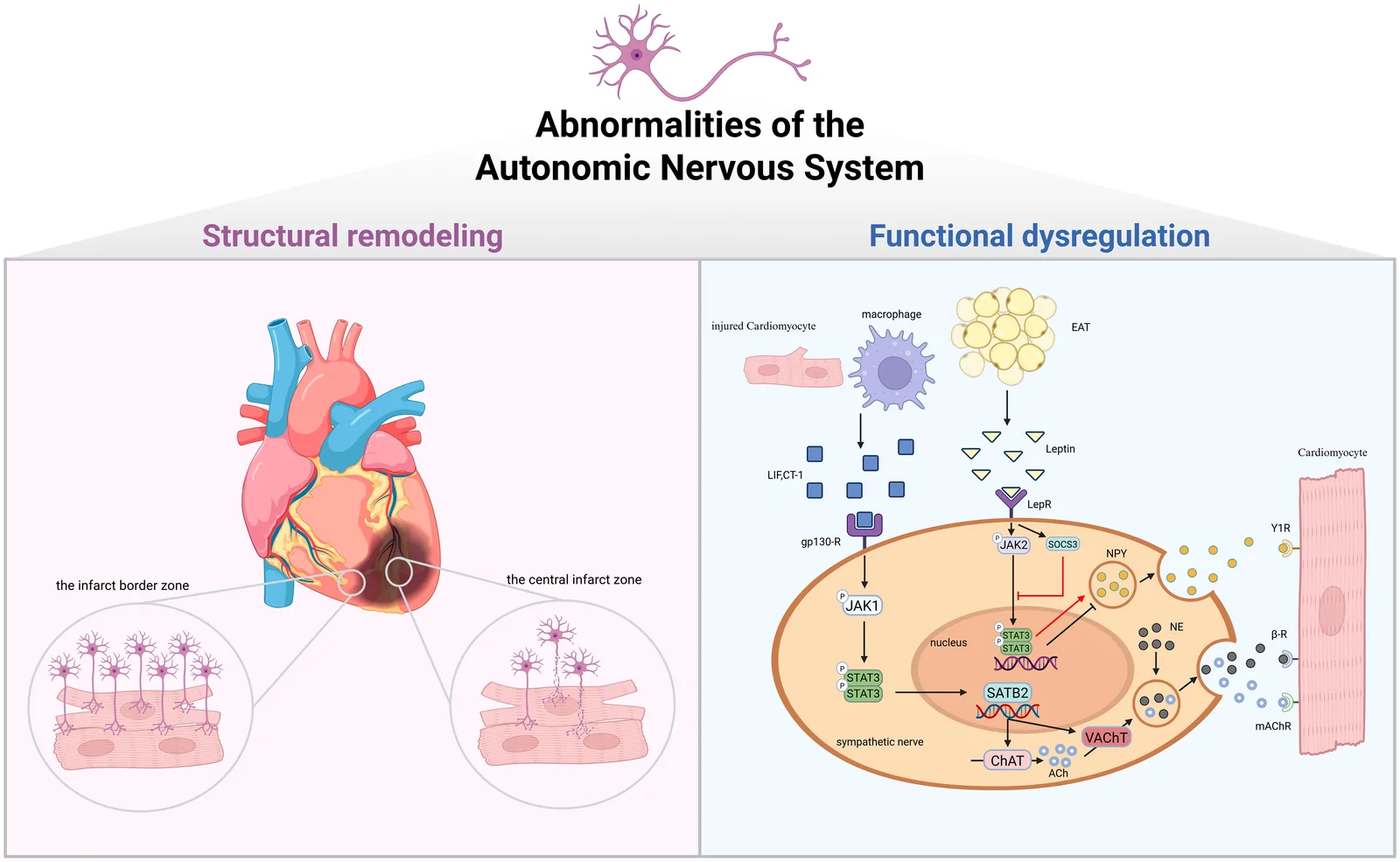
Abnormalities of the autonomic nervous system. In the infarct border zone, nerve regeneration occurs and the density of sympathetic nerve fibers increases. But in the central infarct zone, nerves degenerate and the growth of sympathetic nerve axons are inhibited. This process results in disparities in nerve density between different regions of the heart. Myocardial infarction induces transient cholinergic transdifferentiation of cardiac sympathetic nerves via the gp130 and JAK-STAT signaling pathway. Leptin activates the JAK2-STAT3 pathway in sympathetic neurons via its receptor, upregulates SOCS3 through feedback inhibition, and ultimately leads to substantial neuronal release of NPY. NPY then acts on the Y1 receptor, enhancing NCX activity and activating CaMKII. Figure created with BioRender.com.

Ventricular arrhythmias are frequently attributed to reentry phenomena resulting from asynchronous conduction of sympathetic stimulation, which arises from heterogeneous innervation in the infarct border zone (Koppel et al., 2025). Following myocardial infarction (MI), degeneration of nerves occurs in the central infarct zone, while intense yet disorganized nerve regeneration takes place in the infarct border zone. This process leads to disparities in nerve density across various regions of the heart, thereby establishing heterogeneous innervation. Myocardial scarring is closely associated with abnormal sympathetic nerve distribution (De Bakker et al., 1988). Sulfated chondroitin sulfate proteoglycans present in cardiac scars inhibit the growth of sympathetic nerve axons within the scar tissue, resulting in insufficient sympathetic innervation in these areas (Blake et al., 2022; Gardner and Habecker, 2013). An increase in the density of tyrosine hydroxylase (TH)-positive sympathetic nerve fibers in the border zone of the infarcted myocardium indicates excessive sympathetic innervation (**Figure 1**) (Lyu et al., 2020; Chen et al., 2023). This uneven distribution of sympathetic nerves can lead to variations in action potentials and ion currents, such as the L-type calcium current, across different regions of the heart, resulting in inconsistencies in action potential duration and repolarization (Dajani et al., 2023). Such inconsistencies may trigger early afterdepolarizations and T-wave alternans, thereby initiating arrhythmias.

#### 1.2.2 The autonomic imbalance: functional dysregulation in sympathetic and vagal control

Under physiological conditions, the extrinsic and intrinsic cardiac nervous systems dynamically maintain electrophysiological stability. Following myocardial infarction or structural heart disease, persistent sympathetic activation, vagal withdrawal and intrinsic cardiac neural remodeling promote abnormal automaticity, triggered activity and reentry, increasing susceptibility to ventricular and atrial arrhythmias (Ardell and Armour, 2016; Fukuda et al., 2015; Ajijola et al., 2025a).

The autonomic imbalance, characterized by functional dysregulation in sympathetic and vagal control, is recognized as a manifestation of neural remodeling that increases susceptibility to arrhythmic events (Dusi et al., 2020).

Evidence for autonomic remodeling and arrhythmogenesis is derived from complementary experimental models. Cell-based systems are useful for studying ion channels, calcium handling, and neuron–cardiomyocyte signaling, but they cannot reproduce the intact cardiac neural network. Small-animal models facilitate genetic and molecular studies, whereas large-animal models more closely resemble human cardiac electrophysiology and autonomic anatomy (**Table 1**). However, species differences and the absence of complete neural, immune, and hemodynamic interactions limit direct clinical translation. Therefore, findings from any single model should be interpreted cautiously and validated across complementary models and human studies (Clauss et al., 2019; Ajijola et al., 2025a; Ripplinger et al., 2022).

**Table 1:** Representative experimental models for investigating cardiac autonomic remodeling and their translational relevance.

Model category	Representative models	Major applications	Major translational limitations
Cell-based and co-culture models	hiPSC-derived cardiomyocytes (hiPSC-CMs), sympathetic neuron–cardiomyocyte co-culture systems, isolated cardiomyocytes	Elucidation of molecular and cellular mechanisms, including NE/ACh/NPY signaling, β-adrenergic and muscarinic receptor regulation, Ca^2+^ handling, action potential duration (APD), and triggered activities such as early afterdepolarizations (EADs) and delayed afterdepolarizations (DADs).	Lack integrated central–peripheral autonomic reflexes, hemodynamic regulation, immune microenvironment, and the three-dimensional architecture of cardiac innervation.
Ex vivo heart preparations	Langendorff-perfused hearts, isolated atrial and ventricular tissue preparations, optical mapping platforms	Evaluation of electrical conduction, repolarization heterogeneity, effective refractory period (ERP), arrhythmia inducibility, and the acute electrophysiological effects of pharmacological agents or autonomic neurotransmitters.	Unable to reproduce chronic autonomic remodeling, systemic inflammatory responses, or the complex comorbidities encountered in patients.
Rodent models	Mouse and rat models of myocardial infarction (MI), heart failure (HF), rapid pacing, inflammation, or genetic manipulation	Investigation of neuroimmune interactions, causal validation of molecular pathways, and functional assessment of candidate therapeutic targets *in vivo*.	Significant interspecies differences in heart rate, ion channel composition, cardiac size, and autonomic nervous system anatomy limit direct clinical translation.
Large-animal models	Canine, porcine, and rabbit models of myocardial infarction, ischemia, atrial fibrillation (AF), device stimulation, or catheter ablation	Assessment of neural recordings, feasibility of surgical or device-based interventions, optimization of ablation strategies, and long-term rhythm outcomes under clinically relevant conditions.	High cost, limited sample size, and ethical constraints restrict widespread application; findings still cannot fully substitute for evidence derived from human studies.
Human studies	Human cardiac tissues, cardiac imaging, autonomic mapping, implantable device recordings, prospective cohorts, and randomized clinical trials	Provide the highest level of clinical relevance for evaluating therapeutic efficacy, safety, and clinically meaningful cardiovascular outcomes.	Human tissue studies are predominantly cross-sectional, whereas observational clinical studies are susceptible to confounding; confirmation by adequately powered randomized controlled trials (RCTs) and guideline-level evidence remains necessary.

Following myocardial infarction (MI), inflammatory cytokines induce a transient cholinergic transdifferentiation of cardiac sympathetic nerve fibers via the gp130 and JAK-STAT signaling pathway (Kunisada et al., 1996). Consequently, acetylcholine is co-released with norepinephrine (NE) from sympathetic nerves (Olivas et al., 2016). This “mixed signaling” may hinder the heart’s capacity to adapt to elevated heart rates and increase the risk of arrhythmias. A cholinergic Calbindin neuron population has been identified within the murine intracardiac ganglia (Lizot et al., 2026). These neurons display a significantly reduced N-type calcium current density, an elevated excitability threshold, and diminished overall excitability. In the right atrial ganglionated plexus of atrial fibrillation (AF) patients, there is a reduction in cholinergic and dual-phenotype neurons, accompanied by an increase in noradrenergic neurons. The relative increase in noradrenergic output can heighten the excitability of cardiomyocytes and trigger abnormal activity. This phenomenon creates an electrical substrate that is more susceptible to the initiation and maintenance of multiple-wavelet reentry. Circulating norepinephrine (NE) and leptin in epicardial adipose tissue induce the release of neuropeptide Y (NPY) from ventricular sympathetic nerve terminals (van Weperen et al., 2025; Xiao et al., 2022). NPY interacts with Y1 receptors, subsequently enhancing Na+/Ca2+ exchanger (NCX) and Ca2+/calmodulin-dependent protein kinase II (CaMKII) activity, which leads to abnormal intracellular Ca2+ accumulation in cardiomyocytes and results in delayed afterdepolarizations (Fan et al., 2024; Kalla et al., 2020). Furthermore, it limits acetylcholine release and its protective effects by activating Y2 receptors on parasympathetic nerve terminals (**Figure 1**) (Schwertfeger et al., 2004).

### 1.3 Neuroimmune activation: continuous stimulation of cardiac ganglia

**Figure 2:**
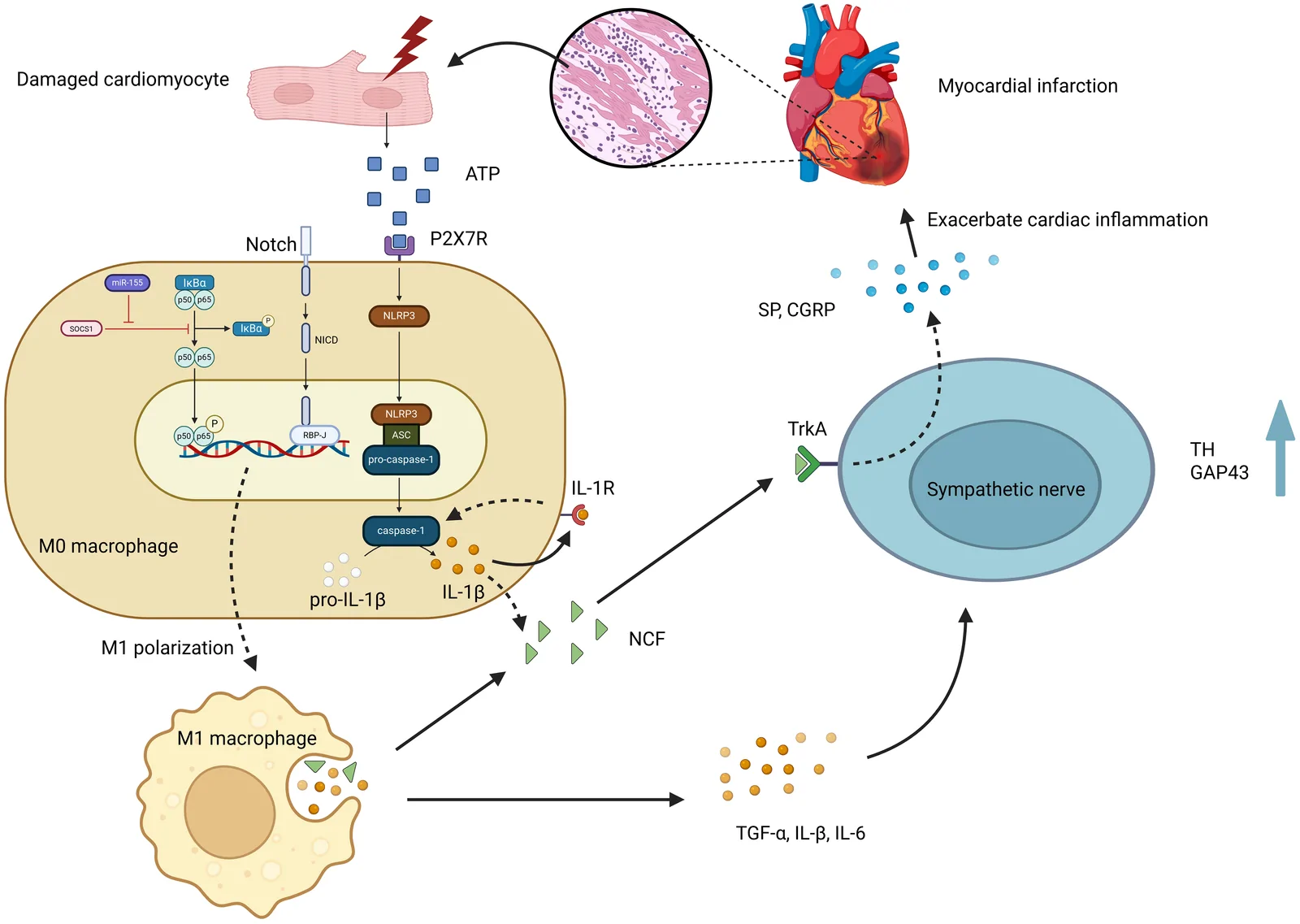
The immunological mechanisms underlying arrhythmias induced by autonomic nervous system dysregulation. Following myocardial infarction, microRNA-155 promotes M1 macrophage polarization and inflammatory responses by targeting and inhibiting SOCS1, thereby activating the NF-κB pathway. This leads to increased expression of NGF, which in turn induces sympathetic neural remodeling and Vas. The activation of the NGF/TrkA signaling pathway and the release of pro-inflammatory cytokines upregulate the expression of TH in neurons, indicating a marked enhancement in their capacity to synthesize and release NE. Concurrently, increased expression of GAP43 reflects active nerve regeneration. NGF signaling further participates in the regulation of neuropeptide synthesis, leading to elevated production of calcitonin gene-related peptide (CGRP) and substance P (SP). These neuropeptides act directly on the local immune milieu, recruiting and activating macrophages, promoting mast cell degranulation, and thereby exacerbating cardiac local inflammation. In addition, the P2X7 receptor contributes to the pathological process of post-myocardial infarction sympathetic nerve sprouting by activating the NLRP3/IL-1β pathway. Activation of the Notch signaling pathway promotes macrophage polarization toward the M1 phenotype and upregulates the expression of NGF. NGF then binds to the TrkA receptors on the growing terminals of sympathetic axons, facilitating neuronal survival, axonal outgrowth, and directional elongation, thereby contributing to sympathetic hyperinnervation. Figure created with BioRender.com.

Hyperactivation of macrophages enhances cardiac sympathetic nerve activity and significantly increases the incidence of ventricular arrhythmias (VAs) (Zhang et al., 2021). Macrophages located in the border zone of infarcted myocardium secrete nerve growth factor (NGF), which promotes sympathetic nerve remodeling (Lyu et al., 2020; Hu et al., 2019). Following myocardial infarction (MI), the expression of NGF derived from macrophages is significantly upregulated in the left superior cervical ganglion (Yu et al., 2025). Additionally, macrophages secrete miR-155, which inhibits suppressor of cytokine signaling 1 (SOCS1), thereby activating the NF-κB pathway. This activation boosts the expression of inflammatory factors and NGF, promoting sympathetic remodeling that leads to arrhythmias (Hu et al., 2019). Furthermore, macrophages facilitate the maturation and release of IL-1β through the P2X7 receptors/NLRP3 inflammasome pathway after MI (Lee et al., 2021; Yin et al., 2017). Subsequently, IL-1β increases susceptibility to atrial fibrillation (AF) by acting on the IL-1 receptor expressed on cardiac resident macrophages, which promotes caspase-1 expression. Activated caspase-1 cleaves pro-IL-1β to generate mature IL-1β, establishing a positive feedback loop that amplifies the local inflammatory response (**Figure 2**) (Moreno-Loaiza et al., 2025). The secretion of NGF can be reduced through macrophage depletion or by blocking associated signaling pathways, such as the SOCS1/NF-κB pathway (Hu et al., 2019) and the NLRP3/IL-1β pathway (Lee et al., 2021; Yin et al., 2017). These interventions lead to the attenuation of sympathetic hyperinnervation and a reduction in the occurrence of arrhythmias (Wernli et al., 2009; Yin et al., 2016).

Other inflammatory factors contribute to ventricular arrhythmias primarily by inducing structural and functional remodeling of sympathetic ganglia, particularly the left stellate ganglion (LSG). Pro-inflammatory cytokines, such as IL-1β (Wang et al., 2017) and IL-17 (Deng et al., 2019), promote the remodeling of the LSG, thereby facilitating the occurrence of ventricular arrhythmias. The superior cervical ganglion (SCG), an extracardiac sympathetic ganglion that regulates cardiac autonomic tone, exhibits significantly upregulated expression of pro-inflammatory genes, including IL-1β and IL-6, following myocardial infarction (MI) (Yu et al., 2025). In contrast, chronic overexpression of the anti-inflammatory cytokine IL-10 within the cardiac sympathetic ganglia (CSG) and LSG markedly suppresses cardiac sympathetic nervous system activity, enhances heart rate variability, reduces LSG function, mitigates post-MI myocardial inflammation, and lowers norepinephrine levels (Li et al., 2024).

## 2 Autonomic modulation therapy for ventricular arrhythmias

**Figure 3:**
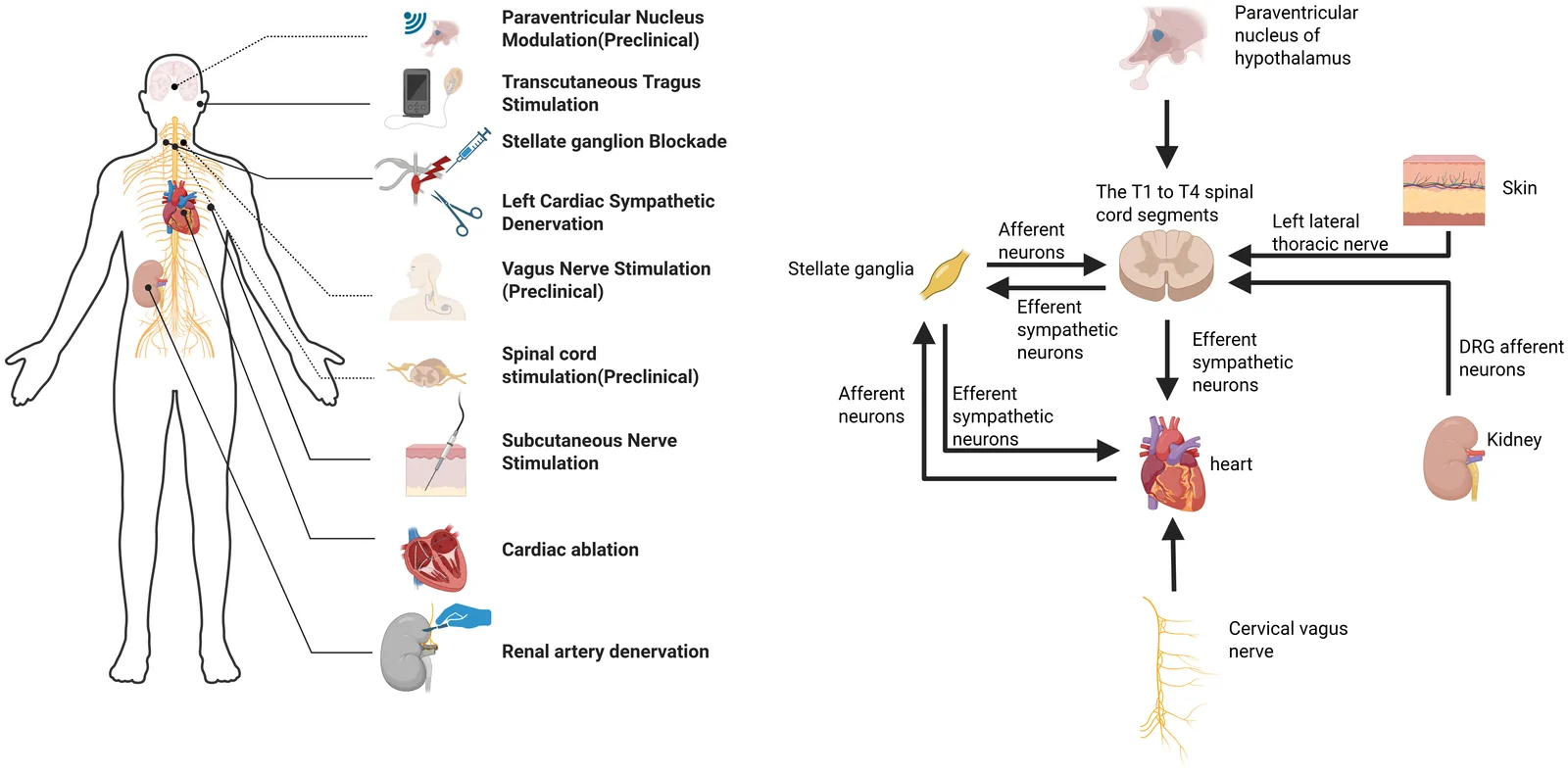
Neuroscientific interventions for cardiac arrhythmia and the targets of autonomic modulation therapy. Figure created with BioRender.com.

Autonomic neuromodulation therapies for ventricular arrhythmias (VAs) encompass a range of approaches, including paraventricular nucleus (PVN) modulation, stellate ganglion blockade (SGB), vagus nerve stimulation (VNS), spinal cord stimulation, cardiac ablation, and renal denervation (RDN) (**Figure 3**). Among these modalities, cardiac ablation and RDN have gained considerable acceptance in clinical practice. These techniques function by modulating the autonomic nervous system (ANS) balance and alleviating excessive sympathetic activation, which in turn reduces the incidence and severity of VAs.

Pharmacological suppression of adrenergic activity remains a central component of clinical arrhythmia management. Beta-blockers, often combined with antiarrhythmic drugs and sedation in electrical storm, reduce sympathetic drive, whereas newer ganglion-targeted ion-channel and anti-inflammatory agents remain investigational.

For clarity, the evidence in each anatomical-target section is interpreted according to its developmental stage. Experimental interventions are presented as mechanistic or proof-of-concept evidence; human case series and non-randomized cohorts are discussed separately as exploratory clinical evidence; and randomized trials, consensus statements, and guideline recommendations are used to define current clinical relevance. This hierarchy is essential because most central, optical, gene-delivery, and targeted ganglionic approaches remain preclinical, whereas SGB and thoracic epidural anesthesia are principally short-term rescue or bridge interventions and cardiac sympathetic denervation is reserved for selected refractory cases.

Less invasive and reversible alternatives to surgical denervation are increasingly being explored. These include pharmacological suppression of sympathetic activity, percutaneous stellate ganglion block, thoracic epidural anesthesia, transcutaneous auricular vagal stimulation, and non-invasive ultrasound-based neuromodulation. Emerging molecular approaches also target ganglionic ion channels, neuroinflammation, and cytokine signaling. However, most molecular, optical, and ultrasound-based strategies remain preclinical, whereas SGB and thoracic epidural anesthesia are currently used mainly as temporary rescue or bridge interventions rather than definitive treatments.

Within each therapeutic subsection, experimental and clinical evidence are presented separately to avoid assigning equivalent weight to preclinical and human findings.

### 2.1 Central autonomic targets

#### 2.1.1 Transcranial ultrasound stimulation

The PVN ultrasound and sonosensitizer studies summarized below are exclusively preclinical. They establish biological plausibility in rodent heart-failure or ischemia models but do not establish human antiarrhythmic efficacy, optimal acoustic parameters, long-term neural safety, or clinical feasibility.

Transcranial ultrasound stimulation can effectively modulate the PVN of the hypothalamus. Low-intensity pulsed ultrasound stimulation (1.0 MHz, 10 Hz, 20% duty cycle, 30 mW/cm²) of the PVN has been shown to suppress P2X7/NLRP3 signaling, thereby preventing heart failure (HF)-induced ventricular arrhythmias (VA) (Yang et al., 2024). The silencing of P2X7 receptors significantly reduces inflammation and sympathetic hyperinnervation (Gao et al., 2017). Additionally, microglia, as resident immune cells in the brain, can be modulated through long-term transcranial ultrasound (1.0 MHz, 2.0 W/cm², 15 min/day for 4 weeks) targeted at the PVN. This intervention regulates the cGAS-STING pathway, promotes microglial polarization toward the M2 phenotype, and mitigates sympathetic overactivation and neuroinflammation (Hu et al., 2025b).

#### 2.1.2 Therapeutic strategies combining sonosensitizers with ultrasound

In contrast to ultrasound stimulation alone, the integration of nanosonosensitizers with ultrasound therapy facilitates mitochondrial targeting and achieves therapeutic goals at reduced ultrasound intensities, owing to the amplifying effect of the sonosensitizers. Mitochondria-targeted sonosensitizers, including BBTD-TPA nanoparticles (Hu et al., 2025a), CCNU980 nanoparticles (Hu et al., 2026), and RuB nanoparticles (Pang et al., 2025), are administered into the paraventricular nucleus (PVN). When subjected to low-intensity focused ultrasound irradiation, these agents produce reactive oxygen species (ROS), which subsequently activate the ROS-AMPK-mTOR signaling pathway. This cascade promotes microglial autophagy and encourages the polarization of microglia toward the M2 phenotype, thereby significantly mitigating sympathetic hyperactivation and neuroinflammation, ultimately leading to a reduction in the incidence of ventricular arrhythmias.

### 2.2 Spinal cord

Experimental evidence. Spinal neuromodulation has shown antiarrhythmic effects mainly in animal models. Epidural stimulation at T1–T4 reduced cardiac sympathetic excitation and ventricular arrhythmias during acute myocardial ischemia in pigs (Howard-Quijano et al., 2017). Intrathecal bupivacaine reduced ventricular premature contractions in ischemic models, whereas optogenetic activation of T1 spinal dorsal horn glutamatergic neurons reduced ventricular tachycardia duration during ischemia and reperfusion (Wu et al., 2022; Omura et al., 2021).Chronic spinal cord stimulation nevertheless requires implanted epidural leads and a pulse generator and carries device- and neuraxial-related risks.

Clinical evidence. Human evidence is currently concentrated on thoracic epidural anesthesia (TEA), whereas spinal cord stimulation, intrathecal anesthesia, and optogenetic spinal modulation remain supported mainly by animal studies. Small observational series and case reports suggest that TEA can rapidly reduce refractory VT/VF burden by blocking thoracic sympathetic outflow, but contemporary electrical-storm consensus documents position it as a specialized and temporary bridge rather than definitive therapy (Do et al., 2017; Lenarczyk et al., 2024).Its use may be restricted by anticoagulation, thrombocytopenia, infection, hypotension, and neuraxial bleeding. Chronic spinal cord stimulation additionally requires epidural lead and pulse-generator implantation and may cause infection, epidural bleeding, neurological injury, lead migration or fracture, and device revision. The predominantly preclinical evidence and procedural risks currently limit routine spinal neuromodulation for ventricular arrhythmias (Howard-Quijano et al., 2017; Koushik et al., 2024).

### 2.3 Extracardiac sympathetic ganglia: stellate ganglion modulation

Targeted ion-channel manipulation, chemical neurolysis, optogenetics, photothermal modulation, and macrophage-directed phototherapy remain experimental. Percutaneous SGB and CT-guided cryoneurolysis have human observational evidence, whereas surgical cardiac sympathetic denervation has the most mature long-term cohort evidence in this target category; nevertheless, randomized comparative trials are still lacking.

Stellate Ganglion Blockade (SGB) diminishes susceptibility to ventricular arrhythmias (VAs) by inhibiting sympathetic activity, which in turn prevents macrophage activation and pro-inflammatory polarization caused by excessive sympathetic excitation.

#### 2.3.1 Ion channel modulation and local anesthetics

Experimental evidence. Ganglion-targeted ion-channel modulation has been explored mainly in animal models. In canine acute myocardial ischemia, microinjection of the M-channel activator retigabine into the left stellate ganglion increased the ventricular fibrillation threshold from 12.33 to 26.67 V (Peng et al., 2025). In rats with acute myocardial infarction, targeted delivery of a CaV2.2 mediator peptide using smart DNA nanoflowers reduced calcium influx and sympathetic overexcitation, thereby improving ventricular electrophysiological stability (Qiao et al., 2025). These studies provide proof-of-concept for local ganglionic ion-channel targeting, but clinical translation remains unproven.

Clinical evidence. Local anesthetic stellate ganglion block using lidocaine, ropivacaine, or bupivacaine is used clinically to suppress sympathetic activity in patients with electrical storm (Patel et al., 2023; Batnyam et al., 2024). Observational series have reported rapid reductions in recurrent ventricular arrhythmias after SGB, although efficacy may be temporary and repeat blockade can be required (Zhang et al., 2025; Motazedian et al., 2024; Fudim et al., 2020). The prospective multicenter STAR study further showed a marked early reduction in treated ventricular arrhythmic events after percutaneous SGB, supporting its use for acute stabilization of refractory electrical storm (Savastano et al., 2024). Nevertheless, its effect may be temporary, and recurrence may require repeat injection or continuous catheter infusion. SGB should therefore be regarded as a rapidly deployable bridge to definitive treatment rather than a therapy for the underlying arrhythmogenic substrate. Available recommendations remain based predominantly on nonrandomized evidence, and procedural risks include vascular puncture, local-anesthetic toxicity, recurrent laryngeal or phrenic nerve block, pneumothorax, and transient Horner syndrome (Lenarczyk et al., 2024; Motazedian et al., 2024).

#### 2.3.2 Electrical blockade of the paravertebral sympathetic chain

The stellate ganglion (SG) is situated within the T1–T2 paravertebral sympathetic chain. Electrical stimulation of the T1–T2 paravertebral sympathetic chain can diminish sympathetic excitation and, as a result, reduce the incidence of ventricular arrhythmias (VAs). In a chronic myocardial infarction (MI) model using Yorkshire pigs, Chui et al. employed charge-balanced direct current carousel block at the T1–T2 segment of the paravertebral sympathetic chain. This intervention decreased the inducibility of ventricular tachycardia (VT) and prolonged the ventricular effective refractory period, thereby enhancing cardiac electrical stability (Chui et al., 2017). Similarly, in a porcine model, Hadaya et al. administered kilohertz frequency alternating current to the right T1–T2 paravertebral sympathetic chain. This approach reversibly and dose-dependently inhibited sympathetic stimulation-evoked cardiac alterations, including increases in heart rate and left ventricular contractility (dP/dtmax) as well as a reduction in the ventricular activation-recovery interval. Furthermore, it significantly decreased norepinephrine (NE) release triggered by sympathetic activation, thus contributing to a reduction in the occurrence of VAs (Hadaya et al., 2022).

#### 2.3.3 Ablation and surgical sympathectomy of SG

Experimental evidence. Experimental studies have investigated targeted ablation of stellate-ganglion sympathetic neurons using chemical and neurotoxin-based approaches. In dogs with chronic myocardial infarction, injection of a cholera toxin B subunit–saporin complex selectively targeted GM1-expressing sympathetic preganglionic neurons and reduced sympathetic activity within the left stellate ganglion (Xiong et al., 2018). In a canine model of acute myocardial infarction, resiniferatoxin selectively ablated transient receptor potential vanilloid 1- and tyrosine hydroxylase-positive neurons in the left stellate ganglion and significantly reduced myocardial-infarction-induced ventricular arrhythmias (Zhou et al., 2019). These studies support the biological feasibility of selective stellate-ganglion neuroablation, but their long-term neural selectivity, reversibility, and clinical safety remain uncertain.

Clinical evidence. Compared with local anesthetic stellate ganglion block, which generally produces temporary sympathetic suppression, cryoneurolysis and surgical cardiac sympathetic denervation may provide more durable effects. Cryoneurolysis produces Sunderland grade II nerve injury, or axonotmesis, and permits subsequent axonal regeneration. In a retrospective single-center study of 17 patients with refractory ventricular arrhythmias, CT-guided left stellate ganglion cryoneurolysis reduced the median number of defibrillations from three to zero within 24 hours. Freedom from defibrillation during the preceding 24 hours was achieved in 82% of patients at 24 hours and 88% at 72 hours, with no reported moderate or high-grade adverse events (Li et al., 2024). However, these findings were derived from a small, uncontrolled cohort and primarily support short-term feasibility rather than definitive efficacy.

Surgical cardiac sympathetic denervation, commonly performed through video-assisted thoracoscopic resection of the lower third of the stellate ganglion and the T2–T4 sympathetic chain, provides a more durable intervention for refractory ventricular arrhythmias. In the largest multicenter cohort, CSD was associated with an 88% reduction in ICD shocks, and 58% of patients remained free from sustained ventricular tachycardia or ICD shocks at one year. Bilateral CSD was associated with better outcomes than left-sided CSD, whereas NYHA class III/IV and longer ventricular-tachycardia cycle length predicted poorer outcomes. An earlier single-center study similarly reported superior shock-free survival after bilateral CSD (Vaseghi et al., 2014, 2017).

Nevertheless, CSD is invasive and largely irreversible. Reported complications include pneumothorax, hemothorax, transient Horner syndrome, hypotension requiring vasopressor support, compensatory hyperhidrosis, and persistent dysesthesia or neuropathic pain (Vaseghi et al., 2014, 2017). CSD should therefore be regarded as a durable rescue or adjunctive strategy for carefully selected patients with recurrent ventricular arrhythmias despite antiarrhythmic therapy and catheter ablation, rather than as an early-line substitute for standard treatment. In contrast, SGB and thoracic epidural anesthesia are more rapidly deployable and reversible and are generally used as short-term bridge interventions (Vaseghi et al., 2014, 2017).

#### 2.3.4 Optical neuromodulation

Recent advances in optical therapy have introduced a novel tool for neurostimulation-based treatment strategies. Inhibition of the SG can be achieved through neuromodulation techniques, including optogenetic modulation, photothermal therapy, and oxygen-independent photodynamic therapy. The gene encoding the inhibitory light-sensitive opsin ArchT was delivered to the LSG neurons of canines using an adeno-associated virus vector, facilitating its expression in sympathetic neurons. Four weeks post-delivery, illumination via a light-emitting diode was administered. This optogenetic modulation was found to reversibly suppress LSG function and neural activity, significantly reduce sympathetic nerve activity, and prolong the left ventricular effective refractory period and action potential duration at 90% repolarization (Yu et al., 2017b). A non-metallic hyperthermia agent, based on aggregation-induced emission luminogen (AIEgen)-based covalent organic frameworks, was injected into the LSG of beagles. Under 808 nm laser irradiation, local hyperthermia therapy was achieved through rapid temperature elevation (Zhang et al., 2025). This method directly suppressed the neural activity of the SG, concurrently induced the browning of white adipose tissue, and improved the neuroinflammatory microenvironment. Platinum nanoshells, possessing high NIR-II photothermal conversion efficiency, mediate precise photothermal modulation to activate the nodose ganglion for parasympathetic enhancement while inhibiting the LSG for sympathetic suppression (Wang et al., 2024). By employing M1 macrophage-targeting oxygen-independent photodynamic therapy, PPS-based nanoparticles were microinjected into the LSG. Under near-infrared light irradiation, the Oi-PDT process generated oxidizing photogenerated holes (h^+^) and reactive oxygen species, resulting in the selective depletion of M1 macrophages. This intervention significantly suppressed sympathetic overexcitation and improved myocardial electrophysiological stability (Lin et al., 2025).

#### 2.3.5 Biological gene therapy targeting the left stellate ganglion

Surgical resection, cryoablation, and chemical blockade of the stellate ganglion (SG) effectively suppress sympathetic overactivation. However, these approaches are invasive, often irreversible, and carry complications such as Horner’s syndrome and pneumothorax (Fudim et al., 2020; Li et al., 2024). Local anesthetics provide only transient effects. These limitations motivate the search for minimally invasive biological alternatives.

The SG functions as a neuroimmune hub where macrophages and cytokines modulate neuronal excitability (Lei et al., 2024). After myocardial infarction, pro-inflammatory signals in the left stellate ganglion (LSG) promote sympathetic hyperinnervation. Conversely, enhancing local anti-inflammatory signaling may interrupt this process. Using adeno-associated virus (AAV)-mediated gene delivery, Li R et al. achieved sustained IL-10 overexpression in the LSG (Li et al., 2024). This intervention reduced tyrosine hydroxylase-positive neurons, downregulated NGF and GAP43, and suppressed sympathetic nerve activity. It prolonged the ventricular effective refractory period and reduced post-infarction ventricular tachycardia/fibrillation (Li et al., 2024). Mechanistically, IL-10 activated the STAT3-DDIT4-mTOR axis (Li et al., 2024). Supporting this concept, Peng et al. showed that AAV-mediated IL-6 overexpression in the LSG exerted opposite pro-arrhythmic effects (Peng et al., 2024). Collectively, these proof-of-concept studies position LSG-targeted gene therapy as a durable, minimally invasive biological alternative to surgical sympathectomy, though safety and translational hurdles remain.

### 2.4 Vagal pathways: vagus nerve stimulation

Two randomized sham-controlled trials have evaluated LLTS for the treatment of frequent premature ventricular complexes (PVCs), with divergent findings. The NoVa-PVC trial was a crossover study enrolling 35 symptomatic patients with a median PVC burden of 12.0% despite β-blocker therapy (Zafeiropoulos et al., 2025). Active LLTS significantly reduced the median PVC burden by 13.4% relative to sham stimulation, with a more pronounced effect in patients with slow heart rate-dependent PVCs. In contrast, the larger TREAT-PVC trial found that both active LLTS and sham stimulation significantly reduced PVC burden, but there was no significant difference between the two groups (Wu et al., 2026). Heart rate variability parameters shifted toward parasympathetic dominance in both arms, while inflammatory markers such as TNF-α, IL-1 and IL-6 and skin sympathetic nerve activity remained unchanged. The TREAT-PVC investigators concluded that the observed PVC reduction was largely attributable to placebo effects and emphasized that future neuromodulation trials must incorporate stringent blinding and account for placebo responses in sample size calculations. The discrepancy between the two trials may reflect differences in study duration, patient selection, or the inherent daily variability of PVC burden. Collectively, these results indicate that while LLTS holds promise as a non-invasive therapy, its efficacy for PVCs remains uncertain, and larger sham-controlled studies with robust blinding are warranted before clinical adoption.

Most evidence for cervical VNS in ventricular arrhythmias derives from canine, porcine, and rodent models. Human data for non-invasive auricular vagal stimulation are limited to small exploratory or randomized crossover studies with surrogate or arrhythmia-burden endpoints; therefore, VNS should not be assigned the same evidentiary weight as established catheter-based therapies.

Vagus nerve stimulation (VNS) can prevent ventricular arrhythmias (VA) by enhancing cardiac mechanical function, stabilizing activation and repolarization in border zones, inhibiting cardiac neuronal sprouting, and suppressing sympathetic hyper-sprouting. VNS effectively counteracts sympathetic activation. Cervical VNS can mitigate the proarrhythmic effects of sympathoexcitation induced by bilateral stimulation of the stellate ganglion and prevent VA and cardiac remodeling without altering heart rate (Hoang et al., 2022; Zhao et al., 2021). HADAYA J et al. evaluated the effects of chronic right cervical VNS on cardiac and extracardiac neural structure and function in Yucatan minipigs following chronic myocardial infarction (MI). They found that chronic VNS improved cardiac mechanical function and reduced the inducibility of post-MI ventricular tachycardia (VT) and ventricular fibrillation (VF) by approximately 85% (Hadaya et al., 2023). In a study conducted by Feng Hu et al., an acute myocardial infarction (AMI) model was established in Sprague-Dawley rats through ligation of the left anterior descending artery. A remotely controlled Vagus Nerve Stimulation (VNS) device was implanted on the right cervical vagus nerve to facilitate unilateral VNS. This intervention significantly reduced the incidence of ventricular arrhythmias (VAs) induced by programmed electrical stimulation, shortened their duration, and improved cardiac function and myocardial fibrosis (Hu F. et al., 2025). Beyond chronic effects, Vaseghi et al. revealed that although infarction reduces central parasympathetic drive, cardiac acetylcholine levels and intrinsic neural pathways remain intact in border zones. Intermittent VNS acutely decreased VT inducibility from 57% to 23% by reducing dispersion of repolarization specifically within the border zone, providing mechanistic evidence for rapid electrical stabilization (Vaseghi et al., 2017b).

Implantable VNS requires cervical vagus nerve dissection, cuff-electrode placement, and subcutaneous generator implantation. Surgical or hardware complications include infection, hematoma, vocal-cord paresis, lead damage, and generator displacement. Stimulation may also cause cough, hoarseness, dysphagia, or bradycardia. These limitations support the development of less invasive auricular stimulation approaches (Révész et al., 2016). In contrast, low-level tragus stimulation modulates autonomic signaling by targeting the auricular branch of the vagus nerve located at the tragus of the outer ear. This method has also exhibited antiarrhythmic effects. Zaffeiro-poulos et al. demonstrated that low-level tragus stimulation in patients with frequent premature ventricular complexes reduced the burden of premature ventricular contractions by exerting antiadrenergic effects, likely through the inhibition of neural activity in the ganglion plexus (GP) and stellate ganglion (SG). A more pronounced response was noted in slow heart rate-dependent premature ventricular contractions, potentially due to the modulation of vagal input, which stabilizes the electrophysiological environment during bradycardia (Zafeiropoulos et al., 2025). Although transcutaneous auricular stimulation avoids an implanted generator and is attractive as a low-risk outpatient approach, its antiarrhythmic efficacy is still supported mainly by small exploratory studies. It should not currently be presented as an established replacement for SGB, TEA, catheter ablation, or CSD in electrical storm.

### 2.5 Intrinsic cardiac neural targets: cardiac ablation

Experimental evidence. Targeted cardiac neural ablation has been evaluated predominantly in animal models. Ablation of neural structures near the ligament of Marshall, coronary sinus, great cardiac vein, and cardiac sympathetic afferent pathways reduced sympathetic activation and ventricular arrhythmia susceptibility (Ma et al., 2018; Chen et al., 2015, 2022). In dogs, pulmonary artery denervation interrupted sympathetic signaling from the left stellate ganglion to the right ventricular outflow tract and reduced sympathetic stimulation-induced ventricular arrhythmias (Zheng et al., 2023).

Clinical evidence. Catheter ablation is established clinically for ventricular tachycardia substrate modification, but its therapeutic benefit should not be attributed solely to autonomic denervation, whose specific contribution remains incompletely quantified. Targeted neural ablation may also be irreversible and carries risks of bleeding, tamponade, vascular or myocardial injury, conduction disturbance, and unintended autonomic damage (Waldron et al., 2019).

### 2.6 Renal sympathetic nerves: renal sympathetic denervation

Experimental evidence. RDN has reduced sympathetic activity and ventricular arrhythmia susceptibility in several animal models. In canine and porcine post-MI models, RDN attenuated cardiac and stellate-ganglion neural remodeling, reduced circulating norepinephrine or myocardial NPY expression, and improved ventricular electrophysiological stability (Yu et al., 2017a; Jackson et al., 2017; Zhang et al., 2018) More recent experimental work also suggests that RDN may suppress ventricular arrhythmogenesis by attenuating macrophage activation and stellate-ganglion neuroinflammation (Hu W. et al., 2025).

Clinical evidence. Direct clinical evidence for RDN in ventricular-arrhythmia prevention remains limited and is derived mainly from small clinical series. Evidence from hypertension indications and blood-pressure trials cannot be directly extrapolated to ventricular-arrhythmia endpoints. Dedicated randomized studies with arrhythmia-specific outcomes are therefore required. Although RDN is less invasive than surgical sympathectomy, it requires arterial access and may cause vascular or contrast-related complications; it should currently remain an investigational adjunct for ventricular arrhythmias (Waldron et al., 2019).

## 3 Autonomic neuromodulation therapy for atrial fibrillation

Autonomic neuromodulatory approaches for atrial fibrillation include subcutaneous or transcutaneous nerve stimulation, ganglionated plexus ablation, pulmonary vein isolation, and renal sympathetic denervation (**Figure 3**). These interventions aim to reduce excessive sympathetic activation and atrial electrical or structural remodeling, but their clinical maturity differs substantially. PVI is an established rhythm-control strategy with collateral autonomic effects, whereas GP ablation, RDN, and low-level tragus stimulation remain adjunctive or investigational approaches whose outcomes may vary according to AF phenotype, hypertension status, procedural context, and study design. Accordingly, mechanistic and animal evidence is presented separately from randomized and observational human evidence in the following subsections.

Autonomic dysregulation contributes to both the initiation and maintenance of AF by promoting triggered activity, spatially heterogeneous refractoriness, and reentry. These effects are particularly relevant in autonomically enriched atrial regions, including the pulmonary veins, posterior left atrium, ligament of Marshall, and ganglionated plexi. This mechanistic link provides the rationale for AF-directed neuromodulatory strategies discussed below (Chen et al., 2014; Ajijola et al., 2025a).

### 3.1 Subcutaneous nerve stimulation

Beyond AF burden, LLTS modulates atrial electrophysiological substrates. Kulkarni et al. reported that chronic LLTS significantly reduced both AF burden and P-wave alternans, a marker of atrial repolarization heterogeneity (Kulkarni et al., 2021). Notably, an acute increase in P-wave alternans during the first LLTS session predicted a favorable long-term response, suggesting P-wave alternans as a potential biomarker for patient selection. Collectively, transcutaneous tragus stimulation is a safe, well-tolerated, non-invasive approach that reduces AF burden and inflammation in paroxysmal AF.

Experimental evidence. ScNS remains supported mainly by experimental and physiological studies. In ambulatory dogs, ScNS reduced sympathetic ganglion activity and showed intensity-dependent antiarrhythmic effects, potentially through suppression of neurite outgrowth and sympathetic hyperinnervation (Wan et al., 2019; Yuan et al., 2020). Physiological studies also demonstrate temporal associations between skin sympathetic nerve activity and short-duration AF or premature atrial contractions (Mao et al., 2024; Hwang et al., 2024).

Clinical evidence. Human evidence in this subsection relates primarily to transcutaneous auricular vagal stimulation rather than implanted ScNS; these interventions should not be regarded as interchangeable. In the randomized sham-controlled TREAT-AF study, chronic low-level tragus stimulation reduced AF burden in patients with paroxysmal AF (Chen et al., 2014; Stavrakis et al., 2020).However, the study was small and single-center, and transcutaneous vagal stimulation should currently be regarded as an exploratory adjunct rather than an established alternative to rhythm-control ablation or antiarrhythmic therapy.

ScNS avoids direct dissection of major autonomic nerves but still requires subcutaneous electrode and generator implantation. Possible device-related limitations include pain, infection, electrode migration, lead failure, and repeat procedures for battery or hardware replacement. Importantly, current evidence is derived mainly from canine studies, and its safety and durability in patients remain unknown (Yuan et al., 2019; Kusayama et al., 2021).

### 3.2 Ganglion plexus ablation

Beyond chemical ablation, catheter-based and surgical GP ablation have also yielded mixed results. The GANGLIA-AF trial randomized patients with paroxysmal AF to selective ectopy-triggering GP ablation (without pulmonary vein isolation, PVI) versus standard PVI (Kim et al., 2022). At 12 months, freedom from ≥30 s atrial arrhythmia was not significantly different between GP ablation and PVI (50% vs. 64%, log-rank P = 0.09). Although GP ablation used less radiofrequency energy and allowed more antiarrhythmic drug discontinuation, it did not achieve non-inferiority to PVI. The AFACT study prospectively randomized patients with advanced AF undergoing thoracoscopic PVI and left atrial lines to additional epicardial GP ablation versus no GP ablation (Driessen et al., 2016). After 1 year, freedom from AF recurrence was similar between groups. Moreover, GP ablation was associated with significantly more major adverse events, including major bleeding, sinus node dysfunction, and pacemaker implantation. Consequently, the study concluded that routine GP ablation should not be performed in these patients. A meta-analysis of radiofrequency GP ablation also confirmed no significant reduction in POAF risk (Consoli et al., 2025). Collectively, available high-level evidence suggests that GP ablation, whether chemical, catheter-based, or surgical, does not consistently improve AF outcomes and may cause harm in certain patient populations. Further research is needed to identify potential responder subgroups before clinical adoption.

Experimental evidence. Chemical or nanoparticle-mediated GP ablation remains primarily preclinical. Nanoformulated calcium chloride (nCaCl_2_) can selectively induce neuronal apoptosis within cardiac ganglionated plexi, modify sympathetic and vagal inputs, and attenuate local inflammation. Experimental studies suggest that this approach may reverse atrial electrical remodeling and reduce AF susceptibility, although its selectivity, durability, and clinical translatability remain uncertain (O’Quinn et al., 2019; Jafree et al., 2025).

Clinical evidence. Human studies have produced inconsistent results. Calcium-mediated GP ablation has shown different effects on postoperative AF depending on the type of cardiac surgery, and radiofrequency GP ablation has not consistently reduced postoperative AF (Zhang et al., 2025) (Consoli et al., 2025).In patients with paroxysmal AF, adding GP ablation to pulmonary vein isolation improved freedom from atrial tachyarrhythmias compared with PVI alone (Katritsis et al., 2013). In contrast, the AFACT trial in patients undergoing thoracoscopic ablation for more advanced AF found no improvement in arrhythmia-free survival and reported more procedure-related adverse events and sinus-node dysfunction (Driessen et al., 2016). These divergent findings suggest that the clinical effect of GP ablation may depend on AF phenotype, procedural approach, GP localization, and lesion extent (Driessen et al., 2016; Chen et al., 2014).

GP ablation is irreversible and may non-selectively damage sympathetic, parasympathetic, and sensory fibers. Catheter-based or thoracoscopic procedures carry risks of bleeding, tamponade, thromboembolism, and collateral tissue injury, while incomplete denervation and subsequent reinnervation may limit durability. Given the inconsistent clinical benefit and procedural risks, GP ablation should be restricted to selected patients or clinical studies rather than used routinely (Driessen et al., 2016).

### 3.3 Pulmonary vein isolation

Experimental and mechanistic evidence. Pulmonary vein isolation (PVI) may influence cardiac autonomic regulation through collateral modulation or injury of neural structures surrounding the pulmonary venous myocardial sleeves and posterior left atrium. Radiofrequency PVI has been associated with reduced sympathetic activity and reverse remodeling of the left atrium (Reant et al., 2005). Different ablation energies may produce distinct autonomic effects. Although radiofrequency and cryoballoon ablation appear to induce broadly comparable neuromodulation, differences have been reported between cryoballoon and hotballoon PVI, including the magnitude and persistence of heart-rate-variability changes (Andrade et al., 2025; Suzuki et al., 2025). Thermal and cryothermal ablation may cause collateral injury to cardiac neural structures, whereas pulsed-field ablation appears to produce less neural injury because of its relative tissue selectivity (**Table 2**) (Guo et al., 2023; Musikantow et al., 2023; Gerstenfeld et al., 2024; Graeger et al., 2025). These findings support an autonomic contribution to the effects of PVI but do not establish autonomic denervation as its principal therapeutic mechanism.

**Table 2:** Clinical trials of autonomic therapies for cardiac arrhythmias.

	Clinical trial	Intervention	Target	Types of Arrhythmia	Trial design	Enrollment	Clinical trial identifier	Primary endpoint	Main outcome	Ref.
1	Transcutaneous Electrical Vagus Nerve Stimulation to Suppress Premature Ventricular Contractions: A Crossover, Randomized Clinical Trial	LLTS	Auricular branch of vagus nerve	Symptomatic premature ventricular complexes with >5% daily burden	Two-center, prospective, sham-controlled, single-blinded, crossover randomized clinical trial	35	NCT05341544	PVC burden during active LLTS versus sham stimulation; safety/tolerability was also assessed.	LLTS produced a greater reduction in median PVC burden than sham (approximately 13.4% vs 8.6%; P = 0.021), without major safety concerns.	(Zafeiropoulos et al., 2025)
2	Transcutaneous Electrical Vagus Nerve Stimulation to Suppress Ventricular Premature Complexes (TREAT-PVC): A Randomized Clinical Trial	LLTS	Auricular branch of vagus nerve	Premature ventricular complexes (burden ≥10%)	Prospective, multicenter, double-blind, sham-controlled, randomized	100 (96 analyzed)	NCT04909528	Change in PVC burden after 6 months of LLTS versus sham stimulation.	PVC burden decreased in both groups, but LLTS was not superior to sham; no significant stimulation-related adverse effects were reported.	(Wu et al., 2026)
3	Using subcutaneous nerve stimulation to control atrial fibrillation	Subcutaneous nerve stimulation (ScNS)	Subcutaneous sympathetic nerves (cervicothoracic region)	Paroxysmal atrial fibrillation	Prospective, randomized, sham-controlled	12 analyzed	NCT04529941	Change in AF burden during active ScNS versus sham stimulation.	The small pilot demonstrated feasibility, but the between-group difference in AF-burden reduction was not definitive; the findings support further adequately powered trials.	(Chen et al., 2026)
4	Cardiovascular Disease-Specific Responses to Autonomic Denervation	Calcium-mediated autonomic denervation	Ganglionated plexi	Postoperative atrial fibrillation	Randomized, double-blind, sham-controlled	160,239	ChiCTR2000029314 (CAP-AF2), ChiCTR2000029313 (CAP-AF3)	Incidence of POAF lasting ≥30 seconds within 7 days after surgery.	Effects were disease/procedure specific: CAP-AF2 was stopped early for safety after an unexpected increase in POAF, whereas CAP-AF3 did not show a consistent preventive benefit.	(Zhang et al., 2025)
5	Ectopy-triggering ganglionated plexuses ablation to prevent atrial fibrillation: GANGLIA-AF study	Ectopy-triggering ganglionated plexus ablation (no PVI)	Ganglionated plexi	Paroxysmal atrial fibrillation	Prospective, randomized, controlled, 3-center trial	102 (per-protocol)	NCT02487654	Freedom from atrial arrhythmia after ectopy-triggering GP ablation versus PVI.	GP ablation without PVI was feasible but did not demonstrate superior arrhythmia-free survival; repeat procedures were more frequent than after PVI.	(Kim et al., 2022)
6	Ganglion Plexus Ablation in Advanced Atrial Fibrillation: The AFACT Study	Thoracoscopic ganglion plexus ablation + PVI	Ganglionated plexi	Advanced atrial fibrillation (paroxysmal and persistent)	Prospective, randomized, controlled	240	NCT01091389	Freedom from recurrent AF at 1 year after thoracoscopic ablation with versus without GP ablation.	Additional GP ablation did not improve 1-year arrhythmia-free survival and increased major bleeding, sinus-node dysfunction, and pacemaker implantation.	(Driessen et al., 2016)
7	Effect of Renal Denervation and Catheter Ablation vs Catheter Ablation Alone on Atrial Fibrillation Recurrence Among Patients With Paroxysmal Atrial Fibrillation and Hypertension: The ERADICATE-AF Randomized Clinical Trial	Pulmonary vein isolation + renal denervation	Renal sympathetic nerves	Paroxysmal atrial fibrillation with hypertension	Multicenter, single-blind, randomized	302	NCT01873352	Freedom from AF, atrial flutter, or atrial tachycardia at 12 months without antiarrhythmic drugs after the blanking period.	PVI plus renal denervation improved 12-month freedom from atrial tachyarrhythmia compared with PVI alone and also improved blood-pressure control.	(Steinberg et al., 2020)
8	A randomized comparison of pulmonary vein isolation with versus without concomitant renal artery denervation in patients with refractory symptomatic atrial fibrillation and resistant hypertension	Pulmonary vein isolation + renal denervation	Renal sympathetic nerves	Refractory symptomatic atrial fibrillation with resistant hypertension	Randomized, double-blind	27	NCT01117025	Freedom from AF recurrence at 12 months after PVI plus renal denervation versus PVI alone.	AF-free survival was higher with combined therapy (69% vs 29%; P = 0.033), accompanied by greater blood-pressure reduction.	(Pokushalov et al., 2012)
9	Long-Term Changes in Atrial Arrhythmia Burden After Renal Denervation Combined With Pulmonary Vein Isolation: SYMPLICITY-AF	Radiofrequency renal denervation + PVI	Renal sympathetic nerves	Atrial fibrillation (paroxysmal and persistent) with uncontrolled hypertension	Prospective, randomized, pilot	70	NCT02064764	Long-term atrial-arrhythmia burden and freedom from recurrence after renal denervation plus PVI versus PVI alone.	Atrial-arrhythmia burden declined over follow-up, but the study did not establish a clear between-group advantage in conventional recurrence-free survival; results were exploratory.	(Chinitz et al., 2024)
10	Ultrasound-Based Renal Sympathetic Denervation as Adjunctive Upstream Therapy During Atrial Fibrillation Ablation: The ULTRA-HFIB Pilot	Ultrasound renal denervation + AF ablation	Renal sympathetic nerves	Paroxysmal/persistent atrial fibrillation with hypertension	Sham-controlled, single-blind, randomized	100	NCT04182620	Single-procedure freedom from AF or atrial flutter at 12 months without antiarrhythmic drugs.	Adjunctive ultrasound renal denervation did not significantly improve the prespecified 12-month rhythm endpoint compared with sham, although the procedure was feasible.	(Whang et al., 2026)
11	TREAT AF (Transcutaneous Electrical Vagus Nerve Stimulation to Suppress Atrial Fibrillation): A Randomized Clinical Trial	LLTS	Auricular branch of vagus nerve	Paroxysmal atrial fibrillation	Sham-controlled, double-blind, randomized	53	NCT02548754	AF burden during the final 2 weeks of the 6-month treatment period.	LLTS significantly reduced AF burden and circulating TNF-α compared with sham stimulation, with no device-related serious adverse events.	(Stavrakis et al., 2020)
12	Autonomic Effects of Pulsed Field vs Thermal Ablation for Treating Atrial Fibrillation: Subanalysis of ADVENT	Pulsed Field Ablation vs Thermal Ablation	Pulmonary Veins	Paroxysmal Atrial Fibrillation	Prospective, single-blind, multicenter randomized comparison	379	NCT04612244	Post-ablation autonomic changes, assessed principally by heart-rate and heart-rate-variability measures.	Pulsed-field ablation produced attenuated autonomic effects compared with thermal ablation; the clinical significance for long-term AF freedom remains uncertain.	(Gerstenfeld et al., 2024)
13	Long-Term Differences in Autonomic Alterations After Cryoballoon vs Radiofrequency Atrial Fibrillation Ablation	Radiofrequency catheter ablation, Cryoballoon ablation	Pulmonary veins	Drug-refractory paroxysmal atrial fibrillation	Multicenter, prospective, parallel-group, single-blind Retrospective cohort study	346	NCT01913522	Long-term change in autonomic indices after cryoballoon versus radiofrequency PVI.	Both techniques altered autonomic tone; cryoballoon ablation produced larger early changes, but differences diminished during follow-up and did not clearly explain rhythm outcomes.	(Andrade et al., 2025)
14	Subclavian Ansae Stimulation on Cardiac Hemodynamics and Electrophysiology in Atrial Fibrillation: A Target for Sympathetic Neuromodulation	Subclavian Ansae Stimulation	Subclavian Ansae	Paroxysmal Atrial Fibrillation	Prospective, single-center feasibility study	17	NCT05133414	Acute changes in cardiac hemodynamics and electrophysiology during graded subclavian-ansae stimulation.	Stimulation elicited reproducible sympathetic hemodynamic and electrophysiological responses, supporting target feasibility; the study was not designed to test long-term antiarrhythmic efficacy.	(Kanthasamy et al., 2025)
15	Efficacy and safety of transcutaneous auricular vagus nerve stimulation for frequent premature ventricular complexes: rationale and design of the TASC-V trial	Transcutaneous auricular vagus nerve stimulation	Auricular branch of vagus nerve	Frequent premature ventricular complexes	Prospective, randomized, parallel-controlled	90 (planned)	NCT04415203	Change in 24-hour PVC burden after the assigned intervention, with safety and symptom/QoL outcomes.	Protocol/design publication; definitive trial results were not reported in the cited article.	(Liu et al., 2024)
16	Rationale and design of the VAST-AF trial: A randomized controlled, blinded, clinical trial to evaluate transcutaneous vagal nerve stimulation for the prevention of persistent atrial fibrillation recurrence	tVNS	Auricular branch of vagus nerve	Persistent atrial fibrillation (after cardioversion)	Randomized, sham-controlled, double-blind	120 (planned)	NCT05833373	Recurrence of persistent AF after cardioversion during follow-up.	Protocol/design publication; no definitive outcome results were available in the cited report.	(Swojanowsky et al., 2024)

Clinical evidence. PVI is the cornerstone lesion set for catheter ablation in selected patients with symptomatic AF and is supported by randomized clinical trials and current AF guidelines. A meta-analysis also suggested that prophylactic PVI performed during cardiac surgery may reduce postoperative AF and AF recurrence at one-year follow-up, although this setting differs from conventional catheter-based PVI for symptomatic AF (Consoli et al., 2025). PVI should therefore be regarded primarily as an established rhythm-control strategy rather than a dedicated autonomic intervention. Its relevance to neuromodulation lies in the close anatomical relationship between the pulmonary veins, posterior left atrium, and cardiac autonomic neural structures, and in the collateral autonomic effects produced by different ablation technologies (Chen et al., 2014; Joglar et al., 2024; Van Gelder et al., 2024).

PVI remains an invasive left atrial procedure requiring venous access, transseptal puncture, catheter manipulation, and periprocedural anticoagulation. Potential complications include vascular bleeding, cardiac tamponade, thromboembolism, pulmonary vein stenosis, phrenic nerve injury, and rare atrioesophageal fistula. Pulmonary vein reconnection or non-pulmonary-vein triggers may also necessitate repeat ablation (Nakhla et al., 2024).

### 3.4 Renal sympathetic denervation

Experimental evidence. Numerous animal studies have examined the preventive effects of renal sympathetic denervation (RDN) on atrial fibrillation (AF) across various disease models, including persistent AF, renal impairment, acute atrial ischemia/infarction, and chronic kidney disease. Bilateral RDN diminished average sympathetic ganglion nerve activity and prompted structural remodeling in the ipsilateral sympathetic ganglion (SG) and brainstem. This remodeling was characterized by significant neuronal and glial cell apoptosis, an increased proportion of tyrosine hydroxylase (TH)-negative ganglion cells, and heightened reactivity of glial fibrillary acidic protein (Tsai et al., 2017). Chronic kidney disease markedly worsens atrial fibrosis (Hohl et al., 2022). RDN also reduced sympathetic nerve growth, cardiac norepinephrine (NE) levels, and intra-atrial fibrosis, while decreasing the complexity of AF (Linz et al., 2015). Furthermore, it lowered the incidence and duration of AF by inhibiting the activation of sympathetic pathways and the renin-angiotensin-aldosterone system (RAAS) (Liang et al., 2015).

Clinical evidence. Clinical evidence for renal sympathetic denervation (RDN) as an adjunctive AF therapy is also heterogeneous. In ERADICATE-AF, adding RDN to catheter ablation improved freedom from AF, atrial flutter, or atrial tachycardia at 12 months in patients with paroxysmal AF and hypertension. However, the trial did not include a sham RDN procedure and its results apply mainly to hypertensive AF populations. A subsequent prospective randomized two-center RDN+AF study in patients with multidrug-resistant arterial hypertension and paroxysmal or persistent AF did not show better rhythm outcomes with concomitant RDN plus AF ablation than AF ablation alone. Taken together, RDN remains a biologically plausible but still investigational adjunct whose value likely depends on blood pressure phenotype, sympathetic tone, renal nerve ablation completeness, and trial design (Steinberg et al., 2020; Kirstein et al., 2023).

## 4 Emerging pharmacological and immunomodulatory strategies and future directions

### 4.1 Systemic and molecular immunomodulatory strategies

Inflammation provides a potential pharmacological target beyond direct neural interruption. Pro-inflammatory cytokines, including IL-1β, IL-6, and TNF, can alter cardiac ion-channel function, calcium handling, and repolarization, thereby increasing arrhythmia susceptibility (Lazzerini et al., 2023, 2017). In a post-myocardial infarction mouse model, IL-1 receptor blockade with anakinra reduced spontaneous ventricular arrhythmias and improved conduction and intracellular Ca^2+^ handling (De Jesus et al., 2017). These findings support anti-inflammatory therapy as a potential adjunct to conventional neuromodulation. However, direct clinical evidence for preventing ventricular arrhythmias remains limited, and systemic cytokine inhibition carries risks of infection and immunosuppression (Lazzerini et al., 2023).

### 4.2 Neuroimmune targets in atrial fibrillation

Local neuroimmune signaling also provides potential therapeutic targets for atrial fibrillation (AF). Cardiomyocyte NLRP3 inflammasome activation promotes abnormal Ca^2+^ handling, shortening of the atrial effective refractory period, structural remodeling, and increased AF susceptibility (Yao et al., 2018; Dobrev et al., 2023). Pharmacological inhibition of NLRP3 signaling therefore represents a promising non-ablative strategy, although current evidence remains predominantly preclinical (Yao et al., 2018; Dobrev et al., 2023). Cardiac resident macrophages may also influence electrophysiology. Macrophages in the atrioventricular node express connexin 43 and electrically couple with cardiomyocytes, demonstrating that immune cells can directly modulate cardiac conduction (Hulsmans et al., 2017). Together, these findings extend therapeutic concepts beyond neuron-centered interventions toward modulation of cardiomyocyte–immune cell interactions (Hulsmans et al., 2017; Dobrev et al., 2023).

### 4.3 Translational challenges and future perspectives

Immunomodulatory approaches should currently be regarded as investigational adjuncts rather than alternatives to established rhythm-control therapies (Dobrev et al., 2023; Lazzerini et al., 2023). Major barriers include incomplete identification of relevant immune-cell subsets, uncertainty regarding treatment timing, systemic immunosuppressive effects, and substantial differences between experimental models and human arrhythmias (Dobrev et al., 2023). Future studies should distinguish local from systemic immunomodulation, define arrhythmia-specific safety and efficacy endpoints, and identify patients with inflammatory or autonomic phenotypes most likely to benefit. Biomarker-guided and target-specific delivery may ultimately allow neuroimmune therapies to complement conventional pharmacological, catheter-based, and neuromodulatory treatments.

## 5 Conclusion

Autonomic neuromodulation provides a mechanistically plausible and increasingly diverse therapeutic framework for cardiac arrhythmias, spanning central autonomic structures, the spinal cord, extracardiac ganglia, vagal pathways, intrinsic cardiac neural networks, and renal sympathetic nerves. Its therapeutic effects reflect not only changes in neural activity but also interactions among autonomic signaling, myocardial electrophysiology, structural remodeling, and immune-cell activation.

However, the strength of evidence remains uneven. PVI and standard catheter ablation are supported by randomized trials and current guidelines for appropriate arrhythmia indications. In contrast, SGB, TEA, and CSD primarily occupy rescue or adjunctive roles based on observational evidence, cohort studies, and expert consensus. RDN, GP ablation, non-invasive vagal stimulation, central ultrasound, optical neuromodulation, gene delivery, and immunomodulation should remain classified as investigational unless supported by indication-specific randomized data. Physiological autonomic changes should not be interpreted as equivalent to clinical efficacy.

Future studies should use standardized arrhythmia and hard clinical endpoints, directly compare emerging interventions with established treatments, and report procedural harms, durability, and long-term outcomes. Further clarification of neuroimmune mechanisms, together with biomarker-guided and target-specific delivery, may enable conventional rhythm-control therapy to be combined with more selective autonomic or immunomodulatory interventions. Such evidence will be essential before the broad biological potential of autonomic neuromodulation can be translated into routine clinical practice.
